# The boy who lived: staged repair of congenital diaphragmatic hernia with esophageal atresia and tracheoesophageal fistula in a 32-week, 1.5 kg infant, and review of the literature

**DOI:** 10.1093/jscr/rjaf333

**Published:** 2025-05-29

**Authors:** John M Woodward, Patricia Corujo Avila, Bobby Mathew, Kathryn D Bass, P Benson Ham 3rd

**Affiliations:** University at Buffalo, Jacobs School of Medicine and Biomedical Sciences, Department of Surgery, 100 High Street, Buffalo, NY 14215, United States; Division of Pediatric Surgery, John R. Oishei Children’s Hospital, 100 High Street, Buffalo NY 14215, United States; University at Buffalo, Jacobs School of Medicine and Biomedical Sciences, Department of Surgery, 100 High Street, Buffalo, NY 14215, United States; Department of Pediatrics, John R. Oishei Children’s Hospital, 100 High Street, Buffalo, NY 14215, United States; Department of Pediatric Surgery, Carilion Roanoke Memorial Hospital, 1906 Belleview Ave Roanoke, VA 24014, United States; University at Buffalo, Jacobs School of Medicine and Biomedical Sciences, Department of Surgery, 100 High Street, Buffalo, NY 14215, United States; Division of Pediatric Surgery, John R. Oishei Children’s Hospital, 100 High Street, Buffalo NY 14215, United States

**Keywords:** congenital diaphragmatic hernia, tracheoesophageal fistula, esophageal atresia, staged surgical approach, improved survival, pediatric surgery

## Abstract

Congenital diaphragmatic hernia (CDH) associated with esophageal atresia (EA) and tracheoesophageal fistula (TEF) is a rare and often fatal combination with reported survival rates of 6%–26%. We aim to analyze the literature on left sided CDH with EA and TEF and report our experience, hypothesizing that delaying right chest approach for EA/TEF repair improves outcomes. We report a case of a 1.5 kg 32-week patient who survived a staged approach of initial CDH repair and abdominal control of TEF with gastrostomy to water seal and vessel loop encircling the gastroesophageal junction followed by EA/TEF repair at 18 days of life. This case report and review of the literature highlights the benefit of a staged surgical approach for left CDH, EA, and TEF; initially proceeding with CDH repair and abdominal control of the TEF first, followed by EA/TEF repair once the patient stabilizes.

## Introduction

Congenital diaphragmatic hernia (CDH) occurs with an overall incidence of 2.3 per 10 000 births [1]. Esophageal atresia (EA) and/or tracheoesophageal fistula (TEF) occur in ~1 in 3500 births [2]. The concurrence of EA with or without TEF in a patient diagnosed with CDH is 0.5% [3]. In this rare population, there is reported survivability ranging from 6.25% to 26.1% in the literature [3, 4]. We report the survival of a pre-term, low-birthweight infant born with both CDH and EA/TEF and describe the rationale behind why the sequence of staged surgical repair we used may benefit this population with a high risk for mortality.

## Case report

A male was born at 32 + 6/7 weeks via cesarean section due to non-reassuring fetal heart tracings to a 35-year-old with routine prenatal care. CDH was antenatally diagnosed on ultrasound with a lung-head-ratio of 2, and polyhydramnios with amniotic fluid index of 27. At birth, the patient weighed 1.545 kg, had appearance, pulse, grimace, activity, and respiration (APGARS) of 3 (1 min) and 7 (5 min), and was intubated in the delivery room. The orogastric tube was unable to pass beyond 10 cm from the lips, and the subsequent X-ray was concerning for EA/TEF. Further workup identified a hypoplastic thumb, 13 ribs bilaterally, a right sixth dysplastic rib, T4 hemivertebrae on the left, a moderate patent ductus arteriosus, and left aortic arch. The patient oxygenated well on mechanical ventilation with synchronized intermittent mandatory ventilation at FiO_2_ 21%, a positive end expiratory pressure of 4 cm H_2_O and was admitted to the Neonatal Intensive Care Unit.

**Figure 1:**
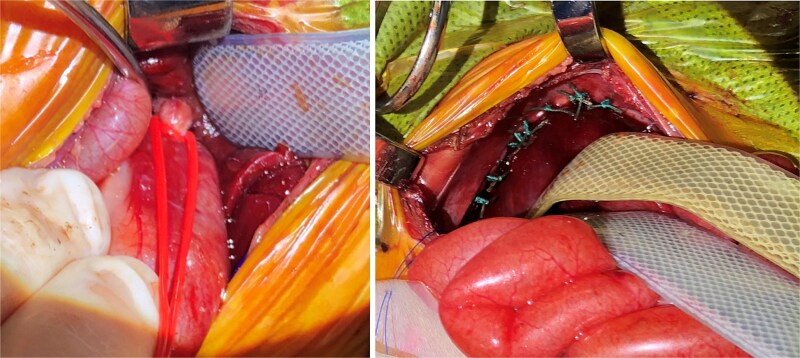
Note (left) the vessel loop around the GE junction in a Potts fashion. This was performed first, followed by CDH repair (right), and then placing a gastrostomy to water seal in the first surgery. Note (right) the distention of the bowels from air shunted through the fistula prior to control of the fistula. After CDH repair and gastrostomy, the vessel loop was loosened and loosely brought out the upper part of the incision at closure and secured.

Approximately 15 h post-delivery the patient proceeded to the operating room. The surgery started with a flexible bronchoscopy which identified a TEF 1 cm above the carina. A repair of the left CDH was then performed though a left upper quadrant laparotomy. First, the stomach was reduced intraabdominally. This was followed by a vessel loop being placed in a Potts fashion encircling the gastroesophageal (GE) junction with the vessel loop then being exteriorized to the skin to allow for the application of additional tension if needed to prevent loss of ventilation through the fistula as shown in Fig. 1. Next, the liver, stomach, spleen, and small bowel were reduced. The diaphragm was closed primarily with interrupted 3–0 Ethibond and a 5 × 5 cm ACELL underlay. Lastly, a 12-Fr gastrostomy tube was placed and put to water seal with a pleuravac, and the laparotomy was closed with the aforementioned vessel loop exteriorized.

Over the next couple weeks, the ventilator was weaned to minimal settings, and a repeat echocardiogram demonstrated decreasing size of the patent ductus arteriosus and improvement in right ventricular pressures and pulmonary hypertension. On hospital day (HD) 18, the patient weighed 1.925 kg and was determined to be ready for the subsequent operation. The patient underwent flexible and rigid bronchoscopy with reidentification of the TEF and visualization of a healthy appearing GE junction and lower esophagus/fistula. A right thoracotomy was performed, the TEF was ligated at the trachea, and the vessel loop removed from the GE junction. A primary esophageal anastomosis was performed and 10F chest tube was placed to complete the operation. On post-operative day (POD)-14, an esophagram showed no leak. On POD-17, gastrostomy tube feeds were started. On POD-21, the patient was extubated to room air, and on POD-23, a repeat esophagram demonstrated esophageal narrowing and reflux.

In the following months, the patient experienced multiple aspiration events and subsequent pneumonia which resulted in multiple reintubations. He otherwise underwent an esophageal dilation on HD 62, and ultimately, on HD 156 was discharged home weighing 4.84 kg and gaining weight on gastrojejunostomy feeds. He has seen significant improvement in his weight from second percentile at his 2-year follow-up to 12th percentile at his 3-year follow-up. The patient continues to follow-up in surgical clinic and demonstrates that he is thriving.

## Discussion

Concurrent EA with TEF and CDH is a condition with high mortality [3–5]. We believe surgical approach has a key role in improving survival for these patients. No uniform approach to left sided CDH associated with EA and TEF exists in the literature. We performed an initial CDH repair with control of the fistula (vessel loop at the GE junction and gastrostomy tube to water seal) followed by EA/TEF repair once the patient’s clinical status had improved, which has been previously performed [6]. There have been other successful approaches including: single-staged approach addressing left CDH, EA, and TEF [7], two-staged repair starting with EA/TEF repair followed by CDH repair [8], and a three-staged approach addressing each congenital abnormality (CDH, TEF, EA) in individual surgeries [5, 9]. This rare constellation of congenital diseases poses unique challenges, and the surgical approach should take into account how the multiple defects interact with each other as well as the clinical status of the child in order to optimize outcomes.

Fifteen left sided CDH with EA and TEF cases with available surgical records to review were identified in the literature (Table 1). Our patient was the smallest by weight (1.545 kg) to survive a two-stage procedure. The only smaller patient, weighing 1.43 kg, underwent a single-staged surgery utilizing an extracorporeal bag for abdominal contents during surgery, followed by later abdominal closure with a patch [7]. These cases demonstrate the survivability of this constellation of congenital diseases in a low-birthweight premature newborn undergoing surgical care. When CDH and TEF ± EA repair were done first and concurrently, 2 of 8 lived (25% survival). However, when initial CDH repair was followed by later delayed TEF ± EA repair 6 of 7 lived (85% survival). We suspect this outcome difference is multifactorial; however, it is why we chose at our institution to proceed with a staged surgical approach of CDH repair first with control of the fistula via gastrostomy to water seal and a loose vessel loop in Potts fashion to control the GE junction.

**Table 1:** Summary of left CDH with associated EA and TEF.

Case report author	Age at birth (weeks)	Weight at birth (kg)	Planned number of surgeries	Surgical order	Survival
Zahn [7]	31	1.32	2	CDH&TEF → EA	No
Zahn [7]	32	1.43	1	CDH&EA&TEF	Yes
Present case	32 6/7	1.55	2	CDH → TEF&EA	Yes
Bagci [10]	33 6/7	1.60	2	CDH → TEF&EA	Yes
Nowicky [11]	33	1.61	2	CDH → TEF&EA	No
Cunát [12]	35 6/7	1.65	1	CDH&TEF&EA	No
Zahn [7]	33	1.67	2	CDH&TEF → EA	No
Charles [6]	34	1.70	2	CDH → TEF&EA	Yes
Abdul Haium [8]	36	1.86	2	TEF&EA → CDH	Yes
Udassin [13]	36	1.90	2	CDH&TEF → Died Before EA	No
Sapin [9]	34	1.95	3	CDH → TEF → EA	Yes
Bösenberg [14]	39	2.14	2	CDH → TEF&EA	Yes
Zahn [7]	35	2.30	2	CDH&TEF → EA	Yes
Zhang [5]	35 3/7	2.35	3	CDH → TEF → EA	Yes
Zahn [7]	36	2.70	2	CDH&TEF → died before EA	No
Al-Salem [15]	39	3.28	1	CDH&EA&TEF	No

Infants with CDH commonly have pulmonary hypoplasia with compensatory pulmonary hypertension. Therefore, in the setting of concurrent CDH and EA/distal TEF, the TEF’s connection to the stomach is a much lower resistance system than the CDH lungs. This can lead to increased preferential ventilation of the fistula rather than the lungs resulting in abdominal distention, decreased diaphragm movement, and worsening pulmonary status resulting in mortality. Early CDH repair, with intra-abdominal fistula control, addresses these concerns and also stays out of the right chest initially. We believe that it is important to advocate for a staged surgical approach in these patients as EA/TEF is typically managed with a right chest approach. This requires right lung compression with reliance on left lung ventilation. With left CDH, depending on the severity, the left lung is often compressed and hypoplastic hindering ventilation. We believe this dynamic can result in insufficient ventilation, pulmonary hypertensive crises, and death. Entering the right chest for repair of the mediastinal defects should be avoided until the patient can tolerate left lung ventilation alone. We suspect the improved survival with the staged approach is due to avoiding pulmonary hypertensive crises and respiratory insufficiency associated with compressing the right lung during early TEF ligation in the chest.

Another innovative approach to improve the compressive effect of the left sided CDH on the left lung was demonstrated by Zahn *et al*. in their single stage approach to treating CDH with associated EA and TEF [7]. They utilized an artificial membrane to exteriorize the herniated contents, improving lung compression, which allowed for appropriate ventilation for this patient while performing the EA/TEF repair through a thoracotomy incision in the right chest at the index operation. We felt this approach would still risk pulmonary hypertensive crisis in our patient.

Additionally, we should mention, that our patient despite being a very low birthweight, premature infant, did not have significant cardiac congenital abnormalities in comparison to other patients which likely improved the ability for this patient to survive surgical repair.

This report and review of the literature highlight the ability for low birth weight premature newborns with left CDH and EA/TEF to survive via staged surgical approach with initial CDH repair and abdominal control of TEF with placement of a gastrostomy to water seal and vessel loop loosely encircling the GE junction. This can help avoid the initial right lung compression of an early right chest surgical approach that can lead to an associated pulmonary hypertensive crisis. The second stage operation is the EA/TEF repair once the patient’s pulmonary hypertension and ventilator requirements are improved, and the patient can tolerate primarily left lung ventilation. This order of operation appears to result in a higher survival for this patient population.
